# Associations between lifetime history of depression, prenatal *OXTR* DNA methylation and breastfeeding outcomes

**DOI:** 10.1186/s13148-025-02030-8

**Published:** 2025-12-30

**Authors:** Elizabeth C. Braithwaite, Kristine Haftorn, Aurora Oftedal, Ziada Ayorech, Anne Kaasen, Christopher Murgatroyd, Robert Lyle, Christian M. Page, Mona Bekkhus

**Affiliations:** 1 School of Psychology, Faculty of Health and Education, Manchester Metropolitan University, Brooks Building, 53 Bonsall Street, Manchester, M15 6GX UK; 2 Department of Rheumatology, Oslo University Hospital, Oslo, Norway; 3 Division of Mental and Physical Health, Department of Childhood and Families, Norwegian Institute of Public Health, Oslo, Norway; 4 Department of Psychology, PROMENTA Research Center, University of Oslo, Oslo, Norway; 5 Faculty of Health Sciences, Oslo Metropolitan University, Oslo, Norway; 6 Department of Life Sciences, Faculty of Science and Engineering, Manchester Metropolitan University, Manchester, UK; 7 Department of Medical Genetics, Oslo University Hospital, Oslo, Norway; 8 Centre for Fertility and Health, Norwegian Institute of Public Health, Oslo, Norway; 9 Department of Physical Health and Ageing, Division of Mental and Physical Health, Norwegian Institute of Public Health, Oslo, Norway

## Abstract

**Background:**

Women with depression are less likely to breastfeed and experience more breastfeeding challenges. Potential biological mechanisms that link maternal depression with poor breastfeeding outcomes have not been investigated. This study examined associations between lifetime history of depression (LHD), prenatal oxytocin receptor gene (*OXTR*) DNA methylation, and breastfeeding outcomes.

**Results:**

Longitudinal observational data from the Norwegian Mother, Father and Child Cohort Study (MoBa) were analysed (N = 3607). LHD was self-reported during pregnancy (week 15). Blood samples were collected at 16–18 weeks of pregnancy, and DNA methylation was measured using the Illumina Methylation EPIC BeadChip 850 K array. This array includes 22 CpG sites on the *OXTR* gene, which were used in analyses. Mothers self-reported breastfeeding initiation (breastmilk given to child in first month), breastfeeding maintenance (breastmilk given to child for 6 months or more) and breastfeeding problems. Maternal LHD was not associated with *OXTR* DNA methylation. There was some evidence that prenatal *OXTR* DNA methylation was associated with breastfeeding outcomes. There was an association between CpG cg26455676 and breastfeeding maintenance to 6 months (odds ratio = 1.59, 95% confidence intervals = 1.11–2.27, raw *p* = 0.01, adjusted *p* = 0.04). Higher levels of DNA methylation at CpG cg26455676 was associated with a greater likelihood of maintaining breastfeeding to 6 months.

**Conclusions:**

This highly novel study highlights the intriguing possibility that maternal prenatal DNA methylation at genes important for breastfeeding may be associated with breastfeeding experiences. Further understanding of vastly differing breastfeeding experiences would assist with the targeting efforts to support breastfeeding women, particularly those who are vulnerable, such as mothers who have depression.

**Supplementary Information:**

The online version contains supplementary material available at 10.1186/s13148-025-02030-8.

## Background

The World Health Organisation (WHO) recommend that infants are breastfed exclusively for the first 6 months of life because of the many important health benefits to both mother and baby [1]. One group of mothers who have particularly low rates of breastfeeding initiation and maintenance are those with depression [2], though the relationship between breastfeeding and depression is complex [3]. For example, women who have depression are less likely to breastfeed, and those who do, produce a lower volume of breastmilk than mothers without depression [4]. There is also evidence that breastfeeding can have a positive impact on maternal mood in the postnatal period [2, 5], and that women with no history of depression during pregnancy who do not initiate breastfeeding are at a greater risk of developing postnatal depression [5]. Thus, there are likely bi-directional relationships between breastfeeding and maternal depression. Here, we focus on the impact of depression on breastfeeding outcomes. Supporting the initiation and maintenance of breastfeeding in women who have symptoms of depression may positively impact maternal mental health.

However, current knowledge on the predictors of breastfeeding initiation and maintenance in women with depression is limited. In terms of the psychological pathways that may explain associations between depression and reduced breastfeeding, there is some evidence that women who have depression report lower breastfeeding self-efficacy [6, 7], are more likely to worry about breastfeeding [8], and are more likely to believe that breastfeeding is not the best feeding method for their child [9]. In addition, women with postnatal depression experience more *physical* difficulties with breastfeeding, including producing a lower volume of breastmilk [4] and self-reporting more breastfeeding problems [10–12]. However, there is currently no knowledge on potential *biological* reasons why women who have depression also have poorer breastfeeding outcomes. One possible biological mechanism which may mediate associations between maternal depression and breastfeeding outcomes may be DNA methylation, which is known to be involved in many biological processes [13]. Evidence from animal models supports the hypothesis that DNA methylation is implicated in milk production [14–17]. Global blood DNA methylation has been shown to be higher in cows with a low milk yield compared with cows with a high milk yield [17], and there is an up-regulation of genes related to fatty acid uptake, synthesis, and secretion during the lactation cycle [16]. Taking inspiration from these findings, the aim of the current study is to investigate whether epigenetic mechanisms may mediate associations between maternal depression and maternal reports of breastfeeding outcomes (initiation, maintenance, and problems).

We chose to focus our investigation on oxytocin: a key hormone associated with the stimulation of milk ejection from the breast and total breastmilk volume in humans [18]. There is evidence that women with depression have impaired milk production and milk ejection, and also changes in breastmilk composition [19], which underscores our rationale to focus on the oxytocin system. The oxytocinergic system is also susceptible to stress-related epigenetic regulation [20], and the gene for the oxytocin receptor (*OXTR*) is epigenetically regulated within individuals in response to stress [20, 21]. Additionally, previous research has demonstrated that mothers with depression have elevated DNA methylation at *OXTR*, which is associated with reduced oxytocin signalling [22].

We have recently demonstrated that maternal lifetime history of depression (LHD) was associated with a lower likelihood of breastfeeding initiation and maintenance, and a greater likelihood of breastfeeding problems [23]. Here, we extend our findings by examining whether A) LHD is associated with *OXTR* DNA methylation, and B) whether *OXTR* DNA methylation is associated with maternal-reported breastfeeding outcomes. We originally developed and pre-registered [24] the following hypotheses:

1. Maternal LHD will be associated with an increase in DNA methylation at *OXTR*.
2. Associations between maternal LHD and a lower likelihood of breastfeeding initiation and maintenance, and a greater likelihood of breastfeeding problems, is mediated by *OXTR* DNA methylation.
  In initial analyses, maternal LHD was not associated with *OXTR* DNA methylation, and therefore we were unable to test hypothesis 2, as *OXTR* DNA methylation is unlikely to mediate associations between lifetime history of depression and breastfeeding outcomes. We therefore developed and tested a third hypothesis:
3. Maternal DNA methylation at *OXTR* will be associated with breastfeeding initiation, maintenance and problems.

## Methods

### Study population

This study used data from the Norwegian Mother, Father and Child Cohort Study (MoBa) [25], a population-based nationwide pregnancy cohort study conducted by the Norwegian Institute of Public Health. Participants were recruited from across Norway between 1999 and 2008, and women consented to participation in 41% of pregnancies [26]. Maternal questionnaire response rates at 15 weeks gestation and 30 weeks gestation were 95.1% and 91.4% respectively [26]. This study was based on version 12 of the quality-assured data files released for research in 2020. In the current study, data from questionnaires at the 15th and 30th week of gestation, as well as 6 months postpartum, were included. We only included data from primiparous mothers (we wanted to focus on first experiences of breastfeeding because of known associations between parity and breastfeeding outcomes [27]), and those mothers with DNA methylation data available. Thus, a total of 3,607 participants from three subsets (met 004, met005 and met008) of MoBa were included in this study: met004 (n = 1615), met005 (n = 851), and met008 (n = 1141). The subsets are reused from previous projects, and all had different study designs; met004 was sampled based on maternal use of ART [28], met005 on advanced maternal age (which included a significant proportion of ART) [29], met008 is a random set among all complete triads in MoBa.

The establishment of MoBa and initial data collection was based on a license from the Norwegian Data Protection Agency and approval from The Regional Committees for Medical and Health Research Ethics. The MoBa cohort is currently regulated by the Norwegian Health Registry Act. The current study was approved by The Regional Committees for Medical and Health Research Ethics (REK:185800).

### Measures

#### Maternal lifetime history of depression

LHD was measured during the second trimester of pregnancy (week 15) by 6 items that closely correspond to the DSM-III criteria for lifetime major depression [30]. Mothers were asked whether they had *ever* experienced each symptom for a period of two weeks or more, and responded with “yes” or “no.” The full wording of all six items is provided in Supplementary Materials. Cronbach’s alpha for the items was 0.85. To meet the criteria for LHD, participants had to endorse the symptom “felt depressed or sad” as well as at least two other symptoms and at least three symptoms had to have been present at the same time.

To address the timing and cause of symptoms, we used two definitions of depression. In the “broad” definition of depression, symptoms could be attributed to external events (e.g., bereavement, divorce), while in the “narrow” definition, such externally caused symptoms were excluded. This allowed us to account for whether symptoms were endogenous or reactive. Whether or not women met the criteria for lifetime depression was coded as a binary variable (yes or no). We have previously used this approach [23].

#### Breastfeeding initiation

Breastfeeding initiation was assessed by maternal self-report at 6 months postpartum. Mothers retrospectively reported how they fed their child during the first month of life. If mothers reported that the infant had received any breast milk during the first month, this was coded as initiation of breastfeeding; otherwise, it was coded as no initiation. Thus, breastfeeding initiation was binary (yes/no).

#### Breastfeeding maintenance

At 6 months postpartum, mothers reported what they had given their child to drink or eat for each month since birth. Among those who initiated breastfeeding, if mothers reported that they had exclusively or partially given breast milk up to and including the sixth month, this was coded as maintaining breastfeeding. If breast milk was reported for 5 months or less, this was coded as not maintaining breastfeeding. Thus, breastfeeding maintenance was binary (yes/no). The 6-month cutoff was chosen in accordance with World Health Organization recommendations for exclusive breastfeeding for the first 6 months of life [1], and this approach has been used in our prior work [23].

#### Breastfeeding problems

Breastfeeding problems were assessed using maternal self-report at 6 months postpartum. Mothers were asked: *“Did you go to your doctor/midwife/health visitor for your own health problems during the first month after the birth?”* with several possible reasons listed, including “breastfeeding problems” (see Supplementary Materials for full wording). Breastfeeding problems were defined as issues other than sore nipples or mastitis, although we also conducted sensitivity analyses including sore nipples in the definition. The breastfeeding problems variable was binary (yes/no). Some mothers who did not initiate breastfeeding, as defined in our study, nevertheless reported breastfeeding problems. This may occur when a mother attempts to breastfeed shortly after delivery but is unable to successfully feed the infant (e.g., due to latching difficulties or insufficient milk production) and therefore does not provide any breast milk during the first month. In such cases, the mother was classified as “not initiating breastfeeding” but was included in the analysis for breastfeeding problems.

#### Confounders

Medical information including method of delivery (vaginal vs caesarean birth), gestational age at birth, child’s sex and maternal age at birth were collected from the Medical Birth Registry of Norway (MBRN) [31]. Birth by caesarean section was registered as yes or no. The length of gestation in days was based on ultrasound estimation, or if ultrasound estimation was not available, gestational length was calculated from the last menstrual period. Child’s sex was coded as male or female. Maternal age at birth was recorded according to the following categories: 24 or younger, 25–29, 30–34, 35–39, 40 or older. Information about maternal education was collected using self-report questionnaires at 15 weeks of gestation. The highest level of completed education was classified according to the following categories: (1) Nine-year secondary school, (2) One-to-two years of high school, (3) Vocational high school, (4) three-year high school general studies, junior college, (5) Regional technical college, four-year university degree (Bachelor’s degree, nurse, teacher, engineer), or (6) University, technical college more than four years (Master’s degree, medical doctor, PhD). Information about maternal smoking was collected using self-report questionnaires during pregnancy at gestational weeks 15 and 30 and was classified according to the following categories: (0) never smoked, (1) smoked, but quit before pregnancy, (2) smoked, but quit early in pregnancy, and (3) continued smoking throughout pregnancy. This was treated as a categorical variable in analyses. For use of medication for depression during pregnancy, women were asked at gestational weeks 15 and 30 if they had taken any medication for depression during weeks 0–4, 5–8. 9–12, 13–16, 17–20, 21–24, 25–28, or 29 + of pregnancy. A positive answer at any timepoint was coded as having taken medication for depression during pregnancy.

Two of the subsets (met004 and met005) were oversampled on mothers who used assisted reproductive technologies (ART) to conceive, thus we adjusted for this in our analyses of these two subsets. Information about the use of ART was collected from MBRN and coded as a binary variable (yes, used ART vs no, did not use ART). In-vitro fertilization and intracytoplasmic sperm injection were defined as ART treatments, whereas intrauterine insemination was not.

### DNAm profiling and quality control

Blood samples for DNAm analyses were collected from the mothers at week 16–18 of pregnancy, or if that was not available, a blood sample taken at birth was used (n = 230). In the MoBa study, biological material was collected at three main timepoints. The first collection took place during weeks 17–20 of pregnancy at the routine ultrasound visit, involving both the pregnant women and the fathers. A few years later, additional funding enabled the collection of two extra blood tubes and a urine sample from the mothers to study environmental exposures; these anaerobically stored whole blood samples are known as K12. Fathers were invited to participate after maternal collections had already begun. The second timepoint was immediately after birth, when biological material was obtained from the newborn’s umbilical cord (or via capillary sampling if needed), alongside new maternal samples collected within 0–3 days postpartum. The Illumina Methylation EPIC BeadChip 850 K array (Illumina, SanDiego, USA) was used for quantifying DNAm. Probes not fulfilling the 5% detection p-value were excluded, and samples with more than 24% missing for the control probes, low bisulfate converstion (< 90%) or above 10% missing were excluded. Background and colour correction was done using out-of-band signals and within-array normalization of type I and II probes was performed using BMIQ from the R package watermelon [32]. Detailed pre-processing steps have been published previously [28]. We analysed 22 CpG sites located in the *OXTR* gene at chromosome three (3p25.3), specific positions are indicated in the results tables.

### Estimation of cell-type proportions

The minfi R package [33] and the FlowSorted.Blood.EPIC reference dataset [34] was used to estimate cell-type proportions for CD4-positive T cells, CD8-postitive T cells, B-cells, granulocytes, monocytes, natural killer cells and nucleated red blood cells from DNAm data in our samples. The estimateCellCounts2 function with Noob for background correction and an iterative algorithm for Identifying Optimal Libraries (IDOL) was used for probe selection. The estimated proportions of each cell type were included as covariates in our analyses.

### Genotyping and mQTL analysis

We used the ARIES methylation quantitative loci (mQTL) database to search for genetic influences on *OXTR* methylation levels [35]. All SNPs associated with any of the CpGs in OXTR from the data base was assessed. Information on the quality control and imputation of the genotypes in MoBa can be found elsewhere [36]. All mothers with DNA methylation in our study was genotyped. One SNP (rs53576) was identified as influencing DNAm levels of two of the *OXTR* CpGs in our dataset (cg00078085 and cg12695586). Output from the ARIES mQTL database search can be found in Supp. Table 1.

### Statistical analysis

Average DNAm levels for each CpG was calculated and visualized using ggplot2 version 3.3.5 [37]. The relation of each CpG to a CpG island was extracted from the Illumina’s Infinium MethylationEPIC v1.0 B5 manifest file. We calculated Pearson correlations between the CpGs and used ggplot2 and reshape2 version 1.4.4 [38] to visualize correlations in a heatmap.

To assess the associations between lifetime history of depression and DNAm levels, DNAm levels for each of the 22 CpGs were regressed on lifetime history of depression using MM-type robust linear regression from the R-package robustbase version 0.93-9 [39], with DNAm as the outcome and lifetime history of depression as the exposure. In our main model, we included maternal smoking, maternal age, selection variable (ART) and cell-type proportions as covariates. In a sensitivity analysis, we included sample timing (when the blood was drawn from the mother) and maternal use of depression medication during pregnancy. Results were reported as M-value effect sizes with standard errors (SE). For analyses on breastfeeding outcomes, we regressed each breastfeeding outcome (initiation, maintenance, and problems), as binary outcomes, on each of the 22 CpGs using logistic regression with a binomial distribution. Results were reported as odds ratios (OR) with 95% confidence intervals (CI). To assess associations with overall *OXTR* methylation we calculated a principal component (PC) score for the 22 CpG sites by running a principal components analysis and extracting the 3 top PCs. These were then included in regression analyses of lifetime history of depression and breastfeeding outcomes in the same framework as the main analyses.

To assess whether differences in genotype might modify the associations (a) between lifetime history of depression and DNAm levels or (b) between DNAm levels and breastfeeding outcomes, we reran the main models including an interaction term between the exposure variable (i.e. model a; lifetime history of depression, and b; DNAm) and the rs53576 genotype. Only cg00078085 and cg12695586 were included in these mQTL analyses.

All analyses were performed in each subset (met004, met005 and met008) separately, and were then meta-analysed using the R-package metafor version 3.0-2 [40]. To control for multiple testing, we applied Bonferroni correction, with a Bonferroni *p* value < 0.05 declared as statistically significant. However, because DNAm levels within a single gene are highly correlated, we used Horn’s parallel analysis to decide how many tests we should correct for [41, 42]. We used the R package paran version 1.5.3 to estimate adjusted eigenvalues for the components [42]. Four components had eigenvalues > 1 and we therefore controlled for four independent tests in the Bonferroni correction. mQTL analyses were not controlled for multiple testing as between the two CpGs only one component had eigenvalue > 1. We used DMRScan to search for differentially methylated regions within the *OXTR* gene [43]. All statistical analyses were performed using R version 4.1.2 [44].

## Results

### Study sample characteristics

Our analyses were based on DNAm data from a total of 3,607 mothers from three subsets of the larger MoBa study: met004 (n = 1615), met005 (n = 851), and met008 (n = 1141). In analyses of breastfeeding outcomes, the sample size was slightly smaller due to missing breastfeeding data at the 6-month follow up (total n = 3117). Characteristics of each subset is shown in Table 1 (LHD analyses) and Table 2 (breastfeeding analyses). The three subsets were relatively similar, but with a few exceptions. Met004 and met005 were oversampled on mothers who used assisted reproductive technologies (ART) to conceive. Furthermore, met005 contained more mothers in the older age groups because the original sampling of this subset only included mothers older than 30 years. 4.3% of the blood samples for DNA methylation in met004 and 19% of the samples in met005 were taken after birth due to methodological limitations of blood sample collection during pregnancy. All samples in met008 were collected from participants during pregnancy.

**Table 1:** Characteristics of samples used for lifetime history of depression and OXTR DNA methylation analyses

Characteristic	met004, N = 1615^*1*^	met005, N = 851^*1*^	met008, N = 1141^*1*^
LHD “broad”, yes	343 (21%)	221 (26%)	238 (21%)
LHD “restrictive”, yes	78 (4.8%)	45 (5.3%)	52 (4.6%)
*Maternal smoking*			
0 (never smoked)	367 (23%)	201 (24%)	214 (19%)
1 (quit before pregnancy)	967 (60%)	520 (61%)	670 (59%)
2 (quit early in pregnancy)	163 (10%)	91 (11%)	168 (15%)
3 (sustained smoking)	118 (7.3%)	39 (4.6%)	89 (7.8%)
*Maternal age (years)*			
< 24	114 (7.1%)	0 (0%)	108 (9.5%)
25–29	459 (28%)	0 (0%)	417 (37%)
30–34	663 (41%)	536 (63%)	432 (38%)
35–39	340 (21%)	289 (34%)	167 (15%)
40 <	39 (2.4%)	26 (3.1%)	17 (1.5%)
*Sample timing*			
Mid pregnancy	1,546 (96%)	690 (81%)	1,141 (100%)
After birth	69 (4.3%)	161 (19%)	0 (0%)
Depression medication, yes	14 (0.9%)	13 (1.5%)	8 (0.7%)
ART, yes	702 (43%)	200 (24%)	2 (0.2%)
*Cell type proportions*			
CD8 + T-cells	0.09 (0.07, 0.12)	0.09 (0.07, 0.11)	0.10 (0.08, 0.12)
CD4 + T-cells	0.09 (0.07, 0.12)	0.09 (0.07, 0.12)	0.09 (0.07, 0.12)
Natural killer cells	0.018 (0.007, 0.031)	0.013 (0.001, 0.027)	0.011 (0.000, 0.026)
B-cells	0.037 (0.028, 0.049)	0.032 (0.022, 0.042)	0.029 (0.020, 0.039)
Monocytes	0.063 (0.051, 0.077)	0.064 (0.051, 0.078)	0.069 (0.057, 0.081)
Granulocytes	0.71 (0.65, 0.76)	0.69 (0.64, 0.74)	0.68 (0.63, 0.73)

^*1*^n (%); Median (IQR), *LHD* Lifetime history of depression, *ART* Assisted reproductive technology

**Table 2:** Characteristics of samples used for OXTR DNA methylation and breastfeeding outcomes analyses

Characteristic		met004, N = 1399^*1*^	met005, N = 744^*1*^	met008, N = 974^*1*^
Breastfeeding initiation, yes		1,274 (91%)	690 (93%)	895 (92%)
Breastfeeding maintenance, yes		1,078 (77%)	607 (82%)	775 (80%)
Breastfeeding problems, yes	Excluding sore nipples	98 (7.6%)	78 (11%)	49 (5.4%)
	Including sore nipples	147 (11.4%)	115 (16.4%)	86 (9.5%)
Excluded*		107	45	73
Maternal smoking				
0 (never smoked)		319 (23%)	176 (24%)	173 (18%)
1 (quit before pregnancy)		843 (60%)	462 (62%)	578 (59%)
2 (quit early in pregnancy)		138 (9.9%)	77 (10%)	149 (15%)
3 (sustained smoking)		99 (7.1%)	29 (3.9%)	74 (7.6%)
Maternal age (years)				
< 24		94 (6.7%)	0 (0%)	76 (7.8%)
25–29		393 (28%)	0 (0%)	353 (36%)
30–34		579 (41%)	472 (63%)	384 (39%)
35–39		297 (21%)	248 (33%)	144 (15%)
40 <		36 (2.6%)	24 (3.2%)	17 (1.7%)
Gestational age at birth (days)		281 (274, 287)	284 (277, 290)	281 (275, 287)
Cesarean section, yes		188 (13%)	114 (15%)	106 (11%)
Maternal education				
1		90 (6.4%)	18 (2.4%)	50 (5.1%)
2		166 (12%)	35 (4.7%)	107 (11%)
3		211 (15%)	72 (9.7%)	143 (15%)
4		588 (42%)	327 (44%)	422 (43%)
5		344 (25%)	292 (39%)	252 (26%)
ART, yes		610 (44%)	174 (23%)	2 (0.2%)
Cell type proportions				
CD8 + T-cells		0.09 (0.07, 0.12)	0.09 (0.07, 0.11)	0.10 (0.08, 0.12)
CD4 + T-cells		0.09 (0.07, 0.12)	0.09 (0.07, 0.12)	0.09 (0.07, 0.12)
Natural killer cells		0.018 (0.008, 0.031)	0.013 (0.001, 0.027)	0.011 (0.000, 0.026)
B-cells		0.037 (0.028, 0.049)	0.031 (0.022, 0.042)	0.029 (0.020, 0.039)
Monocytes		0.063 (0.050, 0.076)	0.065 (0.051, 0.078)	0.069 (0.057, 0.081)
Granulocytes		0.71 (0.65, 0.76)	0.69 (0.64, 0.74)	0.69 (0.63, 0.73)

^*1*^n (%); Median (IQR), *ART* Assisted reproductive technology

*Excluded from analyses of breastfeeding problems, see methods for details

### Characteristics of OXTR CpGs and DNAm

**Fig. 1:**
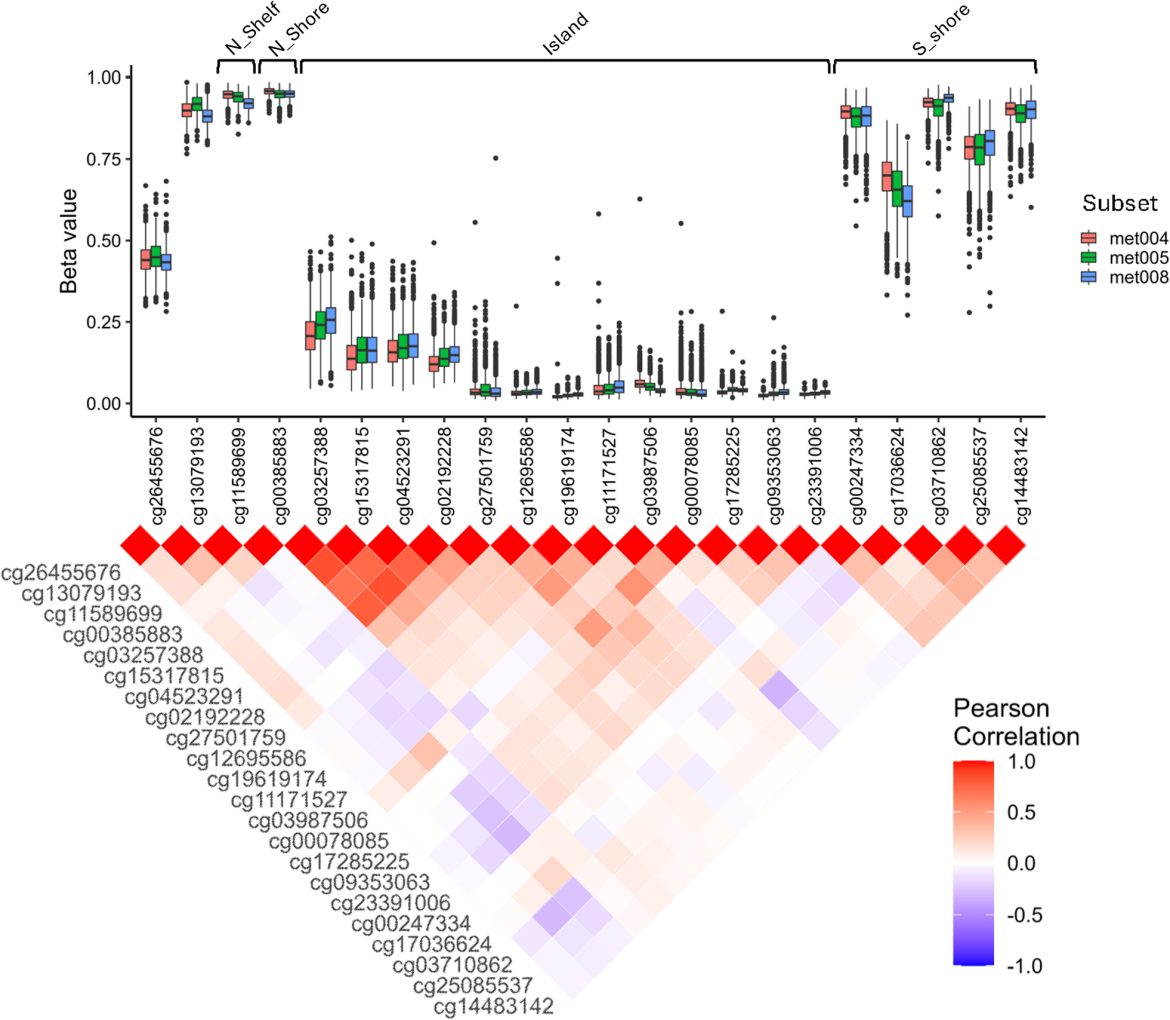
DNA methylation levels and Pearson correlations of 22 CpGs in the *OXTR* gene in three data subsets from MoBa (met004 n = 1615, met005 n = 851, met008 n = 1141). *N_shelf* north shelf, *N_shore* north shore, *S_shore* south shore

We analysed DNAm levels of 22 CpGs located across the *OXTR* gene in all three datasets, where 5 CpGs in the OXTR on the chip were excluded in the QC pipeline. CpGs only present in one of the data sets were excluded. Average DNAm levels across the 22 CpG sites within each dataset are displayed in Fig. 1A. Thirteen of the CpGs were located on a CpG island, five in the south shore, one in the north shore and one in the north shelf of the CpG island. DNAm levels at all CpG sites corresponded well between the three datasets. As expected, CpGs within each region (e.g. island, shore, etc.) were correlated in terms of DNAm levels, especially in the north part of the CpG island and in the south shore, as demonstrated by heatmap Pearson’s correlations in Fig. 1B. A PCA of DNAm levels in the 22 CpGs showed that the three first PCs together explained 39.6–44.5% of the variance in the three datasets (Supp. Fig. 1).

### Associations between LHD and DNA methylation at OXTR

LHD was not associated with DNA methylation at any of the 22 CpGs when using a “broad” definition of LHD (including all causes of depression) as shown in Table 3. When using a more restricted definition of LHD, excluding external causes of depression such as divorce or bereavement (see Methods for details), cg25085537 was negatively associated with LHD (Effect size = − 0.088, *p* = 0.032), but this association was no longer significant after adjusting for multiple testing (Supp. Table 2). Additionally, we found no associations when adjusting for the timing of blood sample collection (Supp. Table 3) or antidepressant medication use during pregnancy (Supp. Table 4). Furthermore, we did not find any evidence of a modifying effect of the rs53576 genotype on the association between LHD and DNAm (Supp. Table 5), nor any indications of differentially methylated regions or associations with overall *OXTR* methylation (Supp. Table 6).

**Table 3:** Results from association tests between LHD and OXTR DNAm levels with adjustments for maternal smoking, maternal age, selection variable (ART), and cell type proportions

CpG ID**	Chr**	Position**	Relation to CpG island**	Effect size (M values)	SE	*p*-value (raw)	*p*-value (adjusted)
cg26455676	3	8,797,459		− 0.01	0.01	0.23	0.93
cg13079193	3	8,802,718		− 0.01	0.02	0.59	1.00
cg11589699	3	8,806,317	N_Shelf	0.00	0.02	0.92	1.00
cg00385883	3	8,808,259	N_Shore	0.00	0.02	0.99	1.00
cg03257388	3	8,809,213	Island	− 0.01	0.02	0.67	1.00
cg15317815	3	8,809,306	Island	0.00	0.03	0.97	1.00
cg04523291	3	8,809,501	Island	− 0.03	0.02	0.24	0.94
cg02192228	3	8,809,536	Island	0.00	0.02	0.83	1.00
cg27501759	3	8,809,715	Island	0.00	0.03	0.94	1.00
cg12695586	3	8,810,077	Island	0.01	0.02	0.63	1.00
cg19619174	3	8,810,139	Island	0.00	0.02	0.89	1.00
cg11171527	3	8,810,206	Island	− 0.01	0.05	0.87	1.00
cg03987506	3	8,810,549	Island	0.00	0.02	0.95	1.00
cg00078085	3	8,810,592	Island	− 0.01	0.02	0.78	1.00
cg17285225	3	8,811,004	Island	0.00	0.01	0.85	1.00
cg09353063	3	8,811,092	Island	0.01	0.02	0.72	1.00
cg23391006	3	8,811,279	Island	− 0.02	0.01	0.08	0.30
cg00247334	3	8,811,543	S_Shore	0.01	0.02	0.80	1.00
cg17036624	3	8,811,601	S_Shore	0.00	0.02	0.84	1.00
cg03710862	3	8,811,728	S_Shore	− 0.02	0.02	0.30	1.00
cg25085537	3	8,811,739	S_Shore	0.00	0.02	0.84	1.00
cg14483142	3	8,811,758	S_Shore	− 0.01	0.03	0.79	1.00

*Chr* Chromosome, *SE* standard error, *N_shelf* north shelf, *N_shore* north shore, *S_shore* south shore

**Information extracted from the Illumina’s Infinium MethylationEPIC v1.0 B5 manifest file. Genomic coordinates are according to the GRCh37 version of the human genome

### Associations between DNA methylation at OXTR and breastfeeding outcomes

We examined associations between maternal DNA methylation at *OXTR* and three breastfeeding outcomes: breastfeeding initiation, breastfeeding maintenance and breastfeeding problems, results are shown in Table 4. After adjustment for multiple testing, cg26455676 alone was associated with breastfeeding maintenance in a positive direction (p_adj_ = 0.04). That is, higher DNA methylation at cg26455676 was associated with a greater likelihood of maintaining breastfeeding to 6 months postpartum. The inclusion of infant gestational age at birth, and delivery by caesarean section in the models did not change the results (see Supp. Table 7). Including maternal education, however, strengthened the association between cg26455676 and breastfeeding maintenance (see Supp. Table 8). Including sore nipples in the definition of breastfeeding problems did not change the results notably (Supp. Table 9). We did not find any evidence of modifying effects of the rs53576 genotype on the association between DNAm and breastfeeding outcomes (Supp. Table 9) nor indications of differentially methylated regions or associations with overall *OXTR* methylation (Supp. Table 6) with any of the breastfeeding outcomes.

**Table 4:** Results from association tests between OXTR DNAm levels and breastfeeding outcomes with adjustment for maternal smoking, maternal age, selection variable (ART), and cell type proportions

Illumina CpG ID	Chr**	Position**	Relation to CpG island**	Model A: Breastfeeding initiation				Model B: Breastfeeding maintenance				Model C: Breastfeeding problems			
				OR	95% CI	*p*-value (raw)	*p*-value (adj)	OR	95% CI	*p*-value (raw)	*p*-value (adj)	OR	95% CI	*p*-value (raw)	*p*-value (adj)
cg26455676	3	8,797,459		1.57	0.93–2.64	0.09	0.36	1.59	1.11–2.27	0.01*	0.04*	0.71	0.36–1.37	0.30	1.00
cg13079193	3	8,802,718		1.21	0.88–1.67	0.25	1.00	1.04	0.86–1.24	0.71	1.00	1.08	0.72–1.63	0.70	1.00
cg11589699	3	8,806,317	N_Shelf	1.07	0.81–1.41	0.65	1.00	1.14	0.93–1.40	0.21	0.84	0.90	0.65–1.25	0.52	1.00
cg00385883	3	8,808,259	N_Shore	0.98	0.73–1.31	0.89	1.00	0.96	0.79–1.17	0.71	1.00	0.86	0.48–1.54	0.62	1.00
cg03257388	3	8,809,213	Island	0.88	0.56–1.37	0.56	1.00	0.91	0.77–1.08	0.27	1.00	0.98	0.76–1.27	0.88	1.00
cg15317815	3	8,809,306	Island	0.88	0.60–1.31	0.54	1.00	0.98	0.86–1.13	0.82	1.00	0.95	0.70–1.29	0.74	1.00
cg04523291	3	8,809,501	Island	0.90	0.50–1.63	0.72	1.00	0.98	0.83–1.15	0.78	1.00	0.94	0.73–1.21	0.63	1.00
cg02192228	3	8,809,536	Island	0.83	0.48–1.44	0.51	1.00	0.96	0.79–1.17	0.71	1.00	1.22	0.90–1.65	0.19	0.77
cg27501759	3	8,809,715	Island	0.90	0.76–1.06	0.21	0.83	0.99	0.88–1.13	0.93	1.00	0.88	0.73–1.07	0.20	0.81
cg12695586	3	8,810,077	Island	0.82	0.49–1.39	0.46	1.00	0.87	0.71–1.07	0.18	0.74	1.07	0.79–1.45	0.67	1.00
cg19619174	3	8,810,139	Island	1.20	0.87–1.64	0.27	1.00	0.97	0.78–1.20	0.78	1.00	0.90	0.63–1.28	0.55	1.00
cg11171527	3	8,810,206	Island	0.94	0.81–1.10	0.43	1.00	0.93	0.84–1.04	0.19	0.75	0.94	0.79–1.12	0.52	1.00
cg03987506	3	8,810,549	Island	1.36	0.98–1.89	0.07	0.27	1.13	0.91–1.41	0.26	1.00	0.81	0.58–1.15	0.25	0.99
cg00078085	3	8,810,592	Island	1.00	0.84–1.19	0.99	1.00	0.98	0.87–1.09	0.67	1.00	1.01	0.84–1.21	0.92	1.00
cg17285225	3	8,811,004	Island	1.14	0.74–1.76	0.55	1.00	0.96	0.71–1.28	0.77	1.00	1.42	0.93–2.17	0.11	0.44
cg09353063	3	8,811,092	Island	1.22	0.87–1.69	0.25	0.98	1.09	0.89–1.34	0.40	1.00	1.03	0.76–1.41	0.83	1.00
cg23391006	3	8,811,279	Island	1.18	0.77–1.80	0.44	1.00	1.14	0.84–1.54	0.42	1.00	0.64	0.41–1.01	0.05	0.21
cg00247334	3	8,811,543	S_Shore	0.75	0.59–0.96	0.02*	0.09	0.92	0.78–1.09	0.35	1.00	1.25	0.97–1.62	0.09	0.35
cg17036624	3	8,811,601	S_Shore	0.83	0.63–1.10	0.20	0.81	0.89	0.74–1.08	0.24	0.95	1.01	0.75–1.35	0.97	1.00
cg03710862	3	8,811,728	S_Shore	0.81	0.55–1.18	0.26	1.00	0.91	0.74–1.12	0.36	1.00	0.99	0.72–1.35	0.93	1.00
cg25085537	3	8,811,739	S_Shore	0.83	0.63–1.08	0.17	0.69	0.82	0.69–0.99	0.04*	0.15	1.35	1.01–1.79	0.04*	0.16
cg14483142	3	8,811,758	S_Shore	0.78	0.62–0.99	0.04*	0.17	0.89	0.76–1.04	0.15	0.60	1.02	0.68–1.52	0.92	1.00

*Chr* Chromosome, *OR* odds ratio, *CI* confidence intervals, *N_shelf* north shelf, *N_shore* north shore, *S_shore* south shore, *adj* adjusted

* Statistically significant* p*-values (< 0.05)

** Information extracted from the Illumina’s Infinium MethylationEPIC v1.0 B5 manifest file. Genomic coordinates are according to the GRCh37 version of the human genome

## Discussion

This study provides a novel examination of the biological underpinnings of breastfeeding challenges in women with a history of depression. Here, we examined whether maternal LHD was associated with maternal DNA methylation at the oxytocin receptor gene (*OXTR*), and whether maternal DNA methylation at the *OXTR* gene was in turn associated with breastfeeding initiation, maintenance, and problems. In models adjusted for potential confounding variables, there was no evidence to suggest that LHD was associated with DNA methylation at the *OXTR* gene. Results remained consistent when using both a broad and narrow definition of LHD. We did however find some evidence to suggest that maternal DNA methylation at the *OXTR* gene was associated with breastfeeding outcomes. The evidence was for an association between the CpG site cg26455676 and the maintenance of breastfeeding to six months after birth. That is, higher levels of maternal DNA methylation at CpG cg26455676 was associated with a greater likelihood of maintaining breastfeeding for at least six months.

These initial findings highlight the intriguing possibility that maternal DNA methylation at genes important for breastfeeding, such as those involved in oxytocin signalling, may be associated with experiences of breastfeeding. As far as the authors are aware, this is the first study of its kind using data from humans, but a recent systematic review has highlighted the role of DNA methylation in the synthesis and secretion of milk fat, milk protein and other nutrients in the milk of cattle, sheep and other mammals [45]. The potential to begin to translate these findings to human research offers opportunities to understand the widely varying breastfeeding experiences of women. A particularly vulnerable group are those with symptoms of postnatal depression, and we know that this group experience greater challenges with breastfeeding: both psychological challenges, such as increased worry and reduced breastfeeding self-efficacy [6–8], but also physical challenges, such as low milk volume and impaired let-down reflex [4, 19]. Although we did not find an association between a lifetime history of depression and DNA methylation at the *OXTR* gene in this study, our examination of OXTR CpG sites was limited by available CpG data from the EPIC BeadChip [46], and other CpG sites on the OXTR gene may play a role in breastfeeding. There is also evidence that DNA methylation at the OXTR promoter region changes across the perinatal period [47], therefore assessments of OXTR DNA methylation during pregnancy may not have utility for predictive associations with postpartum outcomes, and may explain the mostly null findings. Additionally, there are many more maternal genes associated with breastfeeding, including those related to oxytocin and prolactin signalling. Further research to more comprehensively understand whether changes in maternal DNA methylation mediates associations between maternal depression and poor breastfeeding outcomes is a worthy pursuit. Fully understanding biological risk to poor breastfeeding outcomes would help with targeting of efforts to support women who want to breastfeed but may struggle.

Polymorphisms within *OXTR,* including SNP rs53576, have been shown to be associated with depression [48], maternal sensitivity [49], and also aspects of breastfeeding such as occurrence of nipple pain [50]. A recent study reported an interaction between rs53576 and *OXTR* DNA methylation in women who did not have depression prenatally but developed postnatal depression [51]. Furthermore, rs53576 was previously reported to influence DNAm levels of two of the *OXTR* CpGs in the MoBa dataset (cg00078085 and cg12695586) [35]. Therefore, it was important for us to consider possible effects of this polymorphism within this study, though we did not detect any modifying effects.

Strengths of this study include the longitudinal nature of data collection which allowed us to examine, over time, relationships between maternal lifetime history of depression, DNA methylation in pregnancy, and breastfeeding outcomes up to 6 months post-birth. Results of this study, however, should be interpreted in light of the limitations. The sample size, although modest, was limited to MoBa subsets where data on maternal DNA methylation was available. Confidence in the findings presented here would be gained via replication of analyses in more diverse, and larger, samples. Generalisability of our findings to populations outside of European ancestry is challenging because the available MoBa data is limited to the European ancestry subsample. We also recognise that in Norway there are high rates of breastfeeding initiation and maintenance, and that there are large cultural values around breastfeeding which could also contribute to negative feelings when a mother does not breastfeed [52], and therefore the findings presented here may not be generalisable to wider populations. Similarly, only a small number of participants reported LHD and breastfeeding problems, which limited the power of our statistical analysis. A limitation of any human research on epigenetics is the examination of DNA methylation in peripheral tissue (e.g. blood) which may not be representative of DNA methylation in the brain, or mammary tissue [53]. However, we were able to control for cell proportions in our analyses, which is often a limitation of human epigenetic research [53]. Additionally, our analysis of breastfeeding problems was also limited because this was based on maternal report on a single item regarding the presence or absence of breastfeeding problems. A more contextual understanding of maternal DNA methylation linked to specific and detailed breastfeeding problems is an interesting question for future research. It is also important to highlight that breastfeeding is a behaviour to which both the mother and the infant contribute, and it may be in some cases the infant struggles with latching and suckling at the nipple. Therefore, assessments of breastfeeding as a behaviour to which both the mother and child contribute, in relation to the maternal epigenome alone may not be meaningful. A more holistic consideration of the maternal-infant relationship, family environment and wider societal and political influences would be of benefit to this research.

## Conclusion

To conclude, we present a novel investigation of associations between maternal lifetime history of depression, maternal prenatal DNA methylation at the *OXTR* gene, which is linked to breastfeeding and known to be epigenetically regulated under conditions of stress, and breastfeeding outcomes. Initial findings suggest that maternal DNA methylation at a specific CpG site within the *OXTR* gene may be associated with the maintenance of breastfeeding to 6 months post-birth. This lends weight to the intriguing idea that DNA methylation mechanisms may be related to breastfeeding experiences, but much more research with larger and more diverse samples is required before confident conclusions can be drawn.

## Supplementary Information

Supplementary file 1

## Data Availability

The datasets analysed during the current study are available in the MoBa repository, https://www.fhi.no/moba-en
