# Supplementary material for: Associations between lifetime history of depression, prenatal OXTR DNA methylation and breastfeeding outcomes
Source: Clin Epigenetics. 2025 Dec 30;18:14. doi: 10.1186/s13148-025-02030-8 (PMC12817678; doi:10.1186/s13148-025-02030-8)
Supplement: Supplementary file 1 [file 13148_2025_2030_MOESM1_ESM.doc]

**Supp. Table 1. Output from the ARIES mQTL database on SNPs associated with any of the 22 *OXTR* CpGs analysed (search conducted 13.05.2024).**

| **Timepoint** | **SNP** | **SNP Chr** | **SNP Pos** | **A1** | **A2** | **MAF** | **CpG** | **CpG Chr** | **CpG Pos** | **beta** | **t-statistic** | **Effect Size** | **p-value** | **Trans** |
| --- | --- | --- | --- | --- | --- | --- | --- | --- | --- | --- | --- | --- | --- | --- |
| Adolescence | rs53576 | 3 | 8804371 | A | G | 0.318 | cg00078085 | 3 | 8810592 | 0.27299 | 6.31885 | 0.00475 | 4.28e-10 | N |
| Adolescence | rs53576 | 3 | 8804371 | A | G | 0.318 | cg12695586 | 3 | 8810077 | 0.40023 | 8.66878 | 0.00433 | 2.25e-17 | N |
| Childhood | rs53576 | 3 | 8804371 | A | G | 0.321 | cg00078085 | 3 | 8810592 | 0.31991 | 7.97518 | 0.00570 | 5.02e-15 | N |
| Childhood | rs53576 | 3 | 8804371 | A | G | 0.321 | cg12695586 | 3 | 8810077 | 0.34002 | 7.75485 | 0.00429 | 2.58e-14 | N |
| Pregnancy | rs53576 | 3 | 8804371 | A | G | 0.345 | cg00078085 | 3 | 8810592 | 0.25093 | 5.87287 | 0.00189 | 6.39e-09 | N |
| Pregnancy | rs53576 | 3 | 8804371 | A | G | 0.345 | cg12695586 | 3 | 8810077 | 0.30544 | 6.39035 | 0.00400 | 2.88e-10 | N |

*SNP* single nucleotide polymorphism, *Chr* chromosome, *Pos* position, *A1* allele 1, *A2* allele 2, *MAF* minor allele frequency


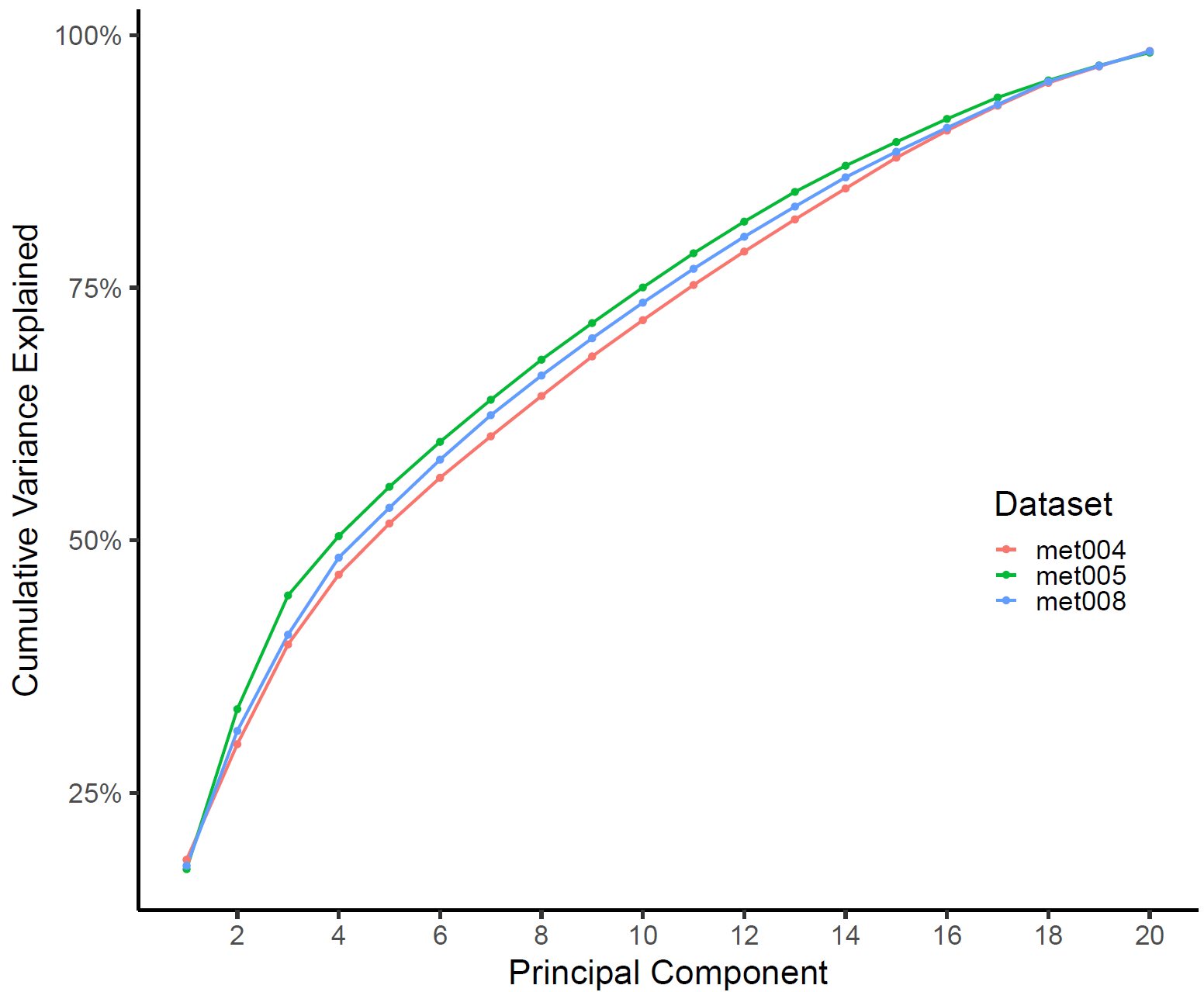


**Supp. Figure 1: Cumulative variance explained by principal components (PCs) 1-20 from PC analyses of DNA methylation levels from 22 *OXTR* CpGs in three MoBa datasets (met004 n = 1,615, met005 n = 851, met008 n = 1,141).**

**Supp. Table 2: Results from association tests between LHD (“restricted” definition) and OXTR DNAm levels with adjustment for maternal smoking, maternal age, cell-type proportions, and selection variable (ART).**

| **Illumina CpG ID** | **Chr**** | **Position**** | **Relation to CpG island**** | **Effect size (M values)** | **SE** | **p-value (raw)** | **p-value (adjusted)** |
| --- | --- | --- | --- | --- | --- | --- | --- |
| cg26455676 | 3 | 8,797,459 |  | 0.00 | 0.02 | 0.89 | 1.00 |
| cg13079193 | 3 | 8,802,718 |  | 0.03 | 0.04 | 0.38 | 1.00 |
| cg11589699 | 3 | 8,806,317 | N_shelf | -0.03 | 0.04 | 0.50 | 1.00 |
| cg00385883 | 3 | 8,808,259 | N_shore | 0.00 | 0.03 | 0.99 | 1.00 |
| cg03257388 | 3 | 8,809,213 | Island | 0.04 | 0.04 | 0.39 | 1.00 |
| cg15317815 | 3 | 8,809,306 | Island | 0.05 | 0.05 | 0.33 | 1.00 |
| cg04523291 | 3 | 8,809,501 | Island | 0.02 | 0.04 | 0.57 | 1.00 |
| cg02192228 | 3 | 8,809,536 | Island | 0.01 | 0.04 | 0.67 | 1.00 |
| cg27501759 | 3 | 8,809,715 | Island | 0.04 | 0.05 | 0.46 | 1.00 |
| cg12695586 | 3 | 8,810,077 | Island | -0.01 | 0.04 | 0.85 | 1.00 |
| cg19619174 | 3 | 8,810,139 | Island | 0.01 | 0.03 | 0.67 | 1.00 |
| cg11171527 | 3 | 8,810,206 | Island | 0.01 | 0.06 | 0.92 | 1.00 |
| cg03987506 | 3 | 8,810,549 | Island | -0.03 | 0.03 | 0.31 | 1.00 |
| cg00078085 | 3 | 8,810,592 | Island | -0.04 | 0.04 | 0.37 | 1.00 |
| cg17285225 | 3 | 8,811,004 | Island | -0.01 | 0.02 | 0.68 | 1.00 |
| cg09353063 | 3 | 8,811,092 | Island | 0.05 | 0.03 | 0.13 | 0.54 |
| cg23391006 | 3 | 8,811,279 | Island | -0.03 | 0.02 | 0.17 | 0.66 |
| cg00247334 | 3 | 8,811,543 | S_shore | -0.04 | 0.09 | 0.67 | 1.00 |
| cg17036624 | 3 | 8,811,601 | S_shore | -0.04 | 0.04 | 0.29 | 1.00 |
| cg03710862 | 3 | 8,811,728 | S_shore | -0.02 | 0.04 | 0.55 | 1.00 |
| cg25085537 | 3 | 8,811,739 | S_shore | -0.09 | 0.04 | **0.03*** | 0.13 |
| cg14483142 | 3 | 8,811,758 | S_shore | -0.04 | 0.04 | 0.31 | 1.00 |

*Chr* Chromosome, *SE* standard error, *N_shelf* north shelf, *N_shore* north shore, *S_shore* south shore
***** Statistically significant p-values (<0.05)
** Information extracted from the Illumina’s Infinium MethylationEPIC v1.0 B5 manifest file. Genomic
coordinates are according to the GRCh37 version of the human genome

**Supp. Table 3: Results from association tests between LHD and *OXTR* DNAm levels with adjustment for maternal smoking, maternal age, selection variable (ART), cell type proportions and sample timing.**

| **Illumina CpG ID** | **Chr **** | **Position**** | **Relation to CpG island**** | **Model A: LHD “broad” definition** | | | | **Model A: LHD “narrow” definition** | | | |
| --- | --- | --- | --- | --- | --- | --- | --- | --- | --- | --- | --- |
|  |  |  |  | **Effect size (M values)** | **SE** | **p-value (raw)** | **p-value (adj)** | **Effect size (M values)** | **SE** | **p-value (raw)** | **p-value (adj)** |
| cg26455676 | 3 | 8797459 |  | -0.01 | 0.01 | 0.37 | 1.00 | 0.00 | 0.03 | 0.91 | 1.00 |
| cg13079193 | 3 | 8802718 |  | -0.03 | 0.03 | 0.30 | 1.00 | 0.05 | 0.05 | 0.37 | 1.00 |
| cg11589699 | 3 | 8806317 | N_shelf | 0.01 | 0.03 | 0.57 | 1.00 | -0.05 | 0.05 | 0.28 | 1.00 |
| cg00385883 | 3 | 8808259 | N_shore | 0.00 | 0.02 | 0.86 | 1.00 | 0.00 | 0.04 | 0.99 | 1.00 |
| cg03257388 | 3 | 8809213 | Island | 0.01 | 0.03 | 0.72 | 1.00 | 0.08 | 0.05 | 0.10 | 0.40 |
| cg15317815 | 3 | 8809306 | Island | 0.01 | 0.03 | 0.85 | 1.00 | 0.10 | 0.07 | 0.13 | 0.53 |
| cg04523291 | 3 | 8809501 | Island | -0.02 | 0.03 | 0.34 | 1.00 | 0.04 | 0.05 | 0.37 | 1.00 |
| cg02192228 | 3 | 8809536 | Island | 0.00 | 0.02 | 0.90 | 1.00 | 0.04 | 0.04 | 0.39 | 1.00 |
| cg27501759 | 3 | 8809715 | Island | 0.01 | 0.03 | 0.66 | 1.00 | 0.06 | 0.06 | 0.33 | 1.00 |
| cg12695586 | 3 | 8810077 | Island | 0.01 | 0.02 | 0.66 | 1.00 | 0.01 | 0.04 | 0.86 | 1.00 |
| cg19619174 | 3 | 8810139 | Island | 0.00 | 0.03 | 0.94 | 1.00 | 0.04 | 0.03 | 0.29 | 1.00 |
| cg11171527 | 3 | 8810206 | Island | -0.04 | 0.05 | 0.41 | 1.00 | 0.01 | 0.07 | 0.84 | 1.00 |
| cg03987506 | 3 | 8810549 | Island | -0.01 | 0.02 | 0.60 | 1.00 | -0.04 | 0.03 | 0.27 | 1.00 |
| cg00078085 | 3 | 8810592 | Island | -0.01 | 0.03 | 0.68 | 1.00 | -0.03 | 0.05 | 0.48 | 1.00 |
| cg17285225 | 3 | 8811004 | Island | 0.00 | 0.01 | 0.95 | 1.00 | -0.01 | 0.04 | 0.89 | 1.00 |
| cg09353063 | 3 | 8811092 | Island | 0.02 | 0.02 | 0.42 | 1.00 | 0.05 | 0.03 | 0.14 | 0.55 |
| cg23391006 | 3 | 8811279 | Island | -0.02 | 0.01 | 0.13 | 0.54 | -0.02 | 0.03 | 0.46 | 1.00 |
| cg00247334 | 3 | 8811543 | S_shore | 0.01 | 0.02 | 0.66 | 1.00 | 0.06 | 0.05 | 0.21 | 0.86 |
| cg17036624 | 3 | 8811601 | S_shore | -0.01 | 0.02 | 0.67 | 1.00 | -0.03 | 0.07 | 0.65 | 1.00 |
| cg03710862 | 3 | 8811728 | S_shore | -0.04 | 0.02 | 0.06 | 0.24 | -0.04 | 0.04 | 0.37 | 1.00 |
| cg25085537 | 3 | 8811739 | S_shore | -0.01 | 0.02 | 0.80 | 1.00 | -0.08 | 0.05 | 0.10 | 0.41 |
| cg14483142 | 3 | 8811758 | S_shore | 0.01 | 0.04 | 0.78 | 1.00 | -0.04 | 0.05 | 0.36 | 1.00 |

*Chr* Chromosome, *SE* standard error, *N_shelf* north shelf, *N_shore* north shore, *S_shore* south shore, *adj* adjusted
** Information extracted from the Illumina’s Infinium MethylationEPIC v1.0 B5 manifest file. Genomic
coordinates are according to the GRCh37 version of the human genome

**Supp. Table 4: Results from association tests between LHD and *OXTR* DNAm levels with adjustment for maternal smoking, maternal age, selection variable (ART), cell type proportions, sample timing and depression medication.**

| **Illumina CpG ID** | **Chr **** | **Position**** | **Relation to CpG island**** | **Model A: LHD “broad” definition** | | | | **Model A: LHD “narrow” definition** | | | |
| --- | --- | --- | --- | --- | --- | --- | --- | --- | --- | --- | --- |
|  |  |  |  | **Effect size (M values)** | **SE** | **p-value (raw)** | **p-value (adj)** | **Effect size (M values)** | **SE** | **p-value (raw)** | **p-value (adj)** |
| cg26455676 | 3 | 8797459 |  | -0.01 | 0.01 | 0.20 | 0.79 | 0.00 | 0.00 | 0.32 | 1.00 |
| cg13079193 | 3 | 8802718 |  | 0.00 | 0.02 | 0.89 | 1.00 | 0.03 | 0.02 | 0.25 | 1.00 |
| cg11589699 | 3 | 8806317 | N_shelf | 0.00 | 0.02 | 0.95 | 1.00 | 0.00 | 0.03 | 1.00 | 1.00 |
| cg00385883 | 3 | 8808259 | N_shore | 0.00 | 0.00 | 0.32 | 1.00 | -0.01 | 0.01 | 0.34 | 1.00 |
| cg03257388 | 3 | 8809213 | Island | 0.00 | 0.02 | 0.92 | 1.00 | 0.03 | 0.05 | 0.56 | 1.00 |
| cg15317815 | 3 | 8809306 | Island | -0.01 | 0.02 | 0.47 | 1.00 | 0.03 | 0.06 | 0.61 | 1.00 |
| cg04523291 | 3 | 8809501 | Island | -0.03 | 0.02 | 0.16 | 0.65 | -0.01 | 0.02 | 0.43 | 1.00 |
| cg02192228 | 3 | 8809536 | Island | -0.01 | 0.01 | 0.33 | 1.00 | -0.01 | 0.02 | 0.60 | 1.00 |
| cg27501759 | 3 | 8809715 | Island | -0.01 | 0.02 | 0.68 | 1.00 | -0.01 | 0.02 | 0.45 | 1.00 |
| cg12695586 | 3 | 8810077 | Island | 0.01 | 0.01 | 0.31 | 1.00 | -0.02 | 0.03 | 0.51 | 1.00 |
| cg19619174 | 3 | 8810139 | Island | 0.00 | 0.01 | 0.93 | 1.00 | 0.00 | 0.03 | 0.97 | 1.00 |
| cg11171527 | 3 | 8810206 | Island | -0.01 | 0.04 | 0.73 | 1.00 | -0.02 | 0.02 | 0.33 | 1.00 |
| cg03987506 | 3 | 8810549 | Island | 0.00 | 0.01 | 0.80 | 1.00 | -0.01 | 0.01 | 0.33 | 1.00 |
| cg00078085 | 3 | 8810592 | Island | 0.01 | 0.01 | 0.65 | 1.00 | -0.04 | 0.04 | 0.28 | 1.00 |
| cg17285225 | 3 | 8811004 | Island | -0.01 | 0.01 | 0.36 | 1.00 | -0.02 | 0.02 | 0.33 | 1.00 |
| cg09353063 | 3 | 8811092 | Island | 0.00 | 0.02 | 0.89 | 1.00 | 0.03 | 0.02 | 0.22 | 0.90 |
| cg23391006 | 3 | 8811279 | Island | -0.02 | 0.01 | 0.08 | 0.34 | -0.03 | 0.03 | 0.26 | 1.00 |
| cg00247334 | 3 | 8811543 | S_shore | 0.00 | 0.02 | 0.91 | 1.00 | 0.09 | 0.04 | 0.05 | 0.20 |
| cg17036624 | 3 | 8811601 | S_shore | 0.00 | 0.00 | 0.31 | 1.00 | -0.03 | 0.05 | 0.51 | 1.00 |
| cg03710862 | 3 | 8811728 | S_shore | -0.01 | 0.02 | 0.57 | 1.00 | 0.00 | 0.00 | 0.31 | 1.00 |
| cg25085537 | 3 | 8811739 | S_shore | 0.00 | 0.00 | 0.31 | 1.00 | -0.08 | 0.05 | 0.10 | 0.38 |
| cg14483142 | 3 | 8811758 | S_shore | 0.00 | 0.03 | 0.98 | 1.00 | -0.03 | 0.03 | 0.27 | 1.00 |

*Chr* Chromosome, *SE* standard error, *N_shelf* north shelf, *N_shore* north shore, *S_shore* south shore, *adj* adjusted
** Information extracted from the Illumina’s Infinium MethylationEPIC v1.0 B5 manifest file. Genomic
coordinates are according to the GRCh37 version of the human genome

**Supp. Table 5. Results from association test between *OXTR* DNAm levels and an interaction term including LHD and rs53576 genotype with adjustment for maternal smoking, maternal age, selection variable (ART), and cell type proportions. Results are reported for the interaction term.**

| **Illumina CpG ID** | **Effect size (M-values)** | **SE** | **p-value (raw)** |
| --- | --- | --- | --- |
| cg12695586 | 0.03 | 0.03 | 0.44 |
| cg00078085 | -0.09 | 0.07 | 0.19 |

*SE* standard error

**Supp. Table 6: Results from regressions between three top principal components (PCs) from PC analyses of DNA methylation levels from 22 *OXTR* CpGs and measures of lifetime history of depression and breastfeeding outcomes.**

| **Principal component** | **Lifetime history of depression*** | | | **Breastfeeding initiation**** | | | **Breastfeeding maintenance**** | | | **Breastfeeding problems**** | | | |
| --- | --- | --- | --- | --- | --- | --- | --- | --- | --- | --- | --- | --- | --- |
|  | **Effect size** | **SE** | **p-value** | **Effect size** | **SE** | **p-value** | **Effect size** | **SE** | **p-value** | **Effect size** | **SE** | **p-value** |  |
| **1** | 0.04 | 0.08 | 0.61 | -0.01 | 0.05 | 0.84 | 0.01 | 0.02 | 0.64 | 0.02 | 0.04 | 0.61 |  |
| **2** | -0.05 | 0.06 | 0.39 | -0.05 | 0.08 | 0.51 | -0.02 | 0.03 | 0.57 | 0.03 | 0.05 | 0.58 |  |
| **3** | -0.05 | 0.06 | 0.37 | 0.04 | 0.08 | 0.65 | -0.01 | 0.05 | 0.75 | 0.06 | 0.05 | 0.19 |  |

All analyses were adjusted for maternal smoking, maternal age, selection variable (ART), and cell type proportions.
* PCs were analysed in separate regression models.
** PCs were analysed together in a multivariate regression model. *SE* standard error

**Supp. Table 7: Results from association tests between *OXTR* DNAm levels and breastfeeding outcomes with adjustment for maternal smoking, maternal age, selection variable (ART), cell type proportions, gestational age, and caesarean section.**

| **Illumina CpG ID** | **Chr**** | **Position**** | **Relation to CpG island**** | **Model A: Breastfeeding initiation** | | | | **Model B: Breastfeeding maintenance** | | | | **Model C: Breastfeeding problems** | | | |
| --- | --- | --- | --- | --- | --- | --- | --- | --- | --- | --- | --- | --- | --- | --- | --- |
|  |  |  |  | **OR** | **95% CI** | **p-value (raw)** | **p-value  (adj)** | **OR** | **95% CI** | **p-value (raw)** | **p-value  (adj)** | **OR** | **95% CI** | **p-value (raw)** | **p-value  (adj)** |
| cg26455676 | 3 | 8797459 |  | 1.57 | 0.93-2.64 | 0.09 | 0.36 | 1.59 | 1.11-2.27 | **0.01*** | **0.04*** | 0.70 | 0.36-1.37 | 0.30 | 1.00 |
| cg13079193 | 3 | 8802718 |  | 1.21 | 0.88-1.67 | 0.25 | 0.99 | 1.04 | 0.86-1.25 | 0.69 | 1.00 | 1.09 | 0.72-1.64 | 0.69 | 1.00 |
| cg11589699 | 3 | 8806317 | N_shelf | 1.07 | 0.80-1.41 | 0.66 | 1.00 | 1.14 | 0.93-1.40 | 0.20 | 0.81 | 0.90 | 0.65-1.25 | 0.52 | 1.00 |
| cg00385883 | 3 | 8808259 | N_shore | 0.98 | 0.74-1.31 | 0.90 | 1.00 | 0.97 | 0.79-1.18 | 0.73 | 1.00 | 0.87 | 0.48-1.58 | 0.65 | 1.00 |
| cg03257388 | 3 | 8809213 | Island | 0.88 | 0.56-1.37 | 0.57 | 1.00 | 0.91 | 0.77-1.08 | 0.28 | 1.00 | 0.98 | 0.76-1.27 | 0.89 | 1.00 |
| cg15317815 | 3 | 8809306 | Island | 0.88 | 0.59-1.32 | 0.55 | 1.00 | 0.98 | 0.86-1.13 | 0.82 | 1.00 | 0.95 | 0.70-1.29 | 0.74 | 1.00 |
| cg04523291 | 3 | 8809501 | Island | 0.90 | 0.50-1.63 | 0.72 | 1.00 | 0.98 | 0.83-1.15 | 0.78 | 1.00 | 0.94 | 0.73-1.22 | 0.65 | 1.00 |
| cg02192228 | 3 | 8809536 | Island | 0.83 | 0.48-1.44 | 0.52 | 1.00 | 0.97 | 0.79-1.18 | 0.73 | 1.00 | 1.23 | 0.91-1.66 | 0.18 | 0.72 |
| cg27501759 | 3 | 8809715 | Island | 0.90 | 0.76-1.06 | 0.21 | 0.84 | 1.00 | 0.88-1.12 | 0.95 | 1.00 | 0.89 | 0.73-1.07 | 0.21 | 0.84 |
| cg12695586 | 3 | 8810077 | Island | 0.82 | 0.49-1.39 | 0.47 | 1.00 | 0.87 | 0.71-1.07 | 0.19 | 0.74 | 1.08 | 0.79-1.46 | 0.64 | 1.00 |
| cg19619174 | 3 | 8810139 | Island | 1.20 | 0.87-1.64 | 0.26 | 1.00 | 0.97 | 0.79-1.20 | 0.80 | 1.00 | 0.90 | 0.63-1.29 | 0.57 | 1.00 |
| cg11171527 | 3 | 8810206 | Island | 0.94 | 0.80-1.10 | 0.42 | 1.00 | 0.93 | 0.83-1.04 | 0.19 | 0.74 | 0.94 | 0.79-1.12 | 0.51 | 1.00 |
| cg03987506 | 3 | 8810549 | Island | 1.35 | 0.97-1.88 | 0.07 | 0.28 | 1.13 | 0.91-1.41 | 0.27 | 1.00 | 0.81 | 0.57-1.15 | 0.23 | 0.93 |
| cg00078085 | 3 | 8810592 | Island | 1.00 | 0.84-1.19 | 0.98 | 1.00 | 0.98 | 0.87-1.09 | 0.67 | 1.00 | 1.01 | 0.84-1.21 | 0.92 | 1.00 |
| cg17285225 | 3 | 8811004 | Island | 1.14 | 0.74-1.76 | 0.55 | 1.00 | 0.95 | 0.71-1.27 | 0.73 | 1.00 | 1.41 | 0.92-2.17 | 0.11 | 0.44 |
| cg09353063 | 3 | 8811092 | Island | 1.22 | 0.87-1.70 | 0.25 | 1.00 | 1.09 | 0.89-1.34 | 0.39 | 1.00 | 1.03 | 0.76-1.41 | 0.83 | 1.00 |
| cg23391006 | 3 | 8811279 | Island | 1.18 | 0.77-1.80 | 0.45 | 1.00 | 1.13 | 0.84-1.52 | 0.43 | 1.00 | 0.64 | 0.40-1.00 | 0.05 | 0.21 |
| cg00247334 | 3 | 8811543 | S_shore | 0.75 | 0.59-0.96 | **0.02*** | 0.09 | 0.93 | 0.78-1.09 | 0.36 | 1.00 | 1.25 | 0.97-1.62 | 0.09 | 0.35 |
| cg17036624 | 3 | 8811601 | S_shore | 0.83 | 0.63-1.10 | 0.20 | 0.81 | 0.89 | 0.74-1.08 | 0.25 | 0.99 | 1.01 | 0.75-1.35 | 0.97 | 1.00 |
| cg03710862 | 3 | 8811728 | S_shore | 0.81 | 0.56-1.17 | 0.26 | 1.00 | 0.90 | 0.73-1.11 | 0.32 | 1.00 | 0.99 | 0.72-1.35 | 0.92 | 1.00 |
| cg25085537 | 3 | 8811739 | S_shore | 0.83 | 0.63-1.08 | 0.17 | 0.69 | 0.82 | 0.68-0.99 | **0.03*** | 0.14 | 1.35 | 1.01-1.79 | **0.04*** | 0.16 |
| cg14483142 | 3 | 8811758 | S_shore | 0.78 | 0.62-0.99 | **0.04*** | 0.17 | 0.89 | 0.76-1.04 | 0.15 | 0.60 | 1.02 | 0.68-1.53 | 0.92 | 1.00 |

*Chr* Chromosome, *SE* standard error, *N_shelf* north shelf, *N_shore* north shore, *S_shore* south shore, *adj* adjusted
***** Statistically significant p-values (>0.05)
** Information extracted from the Illumina’s Infinium MethylationEPIC v1.0 B5 manifest file. Genomic coordinates are according to the GRCh37 version of the human genome

**Supp. Table 8: Results from association tests between *OXTR* DNAm levels and breastfeeding outcomes with adjustment for maternal smoking, maternal age, selection variable (ART), cell type proportions, gestational age, caesarean section, and maternal education.**

| **Illumina CpG ID** | **Chr **** | **Position **** | **Relation to CpG island**** | **Model A: Breastfeeding initiation** | | | | **Model B: Breastfeeding maintenance** | | | | **Model C: Breastfeeding problems** | | | |
| --- | --- | --- | --- | --- | --- | --- | --- | --- | --- | --- | --- | --- | --- | --- | --- |
|  |  |  |  | **OR** | **95% CI** | **p-value (raw)** | **p-value  (adj)** | **OR** | **95% CI** | **p-value (raw)** | **p-value  (adj)** | **OR** | **95% CI** | **p-value (raw)** | **p-value  (adj)** |
| cg26455676 | 3 | 8797459 |  | 1.59 | 0.93-2.70 | 0.09 | 0.35 | 1.65 | 1.15-2.38 | **0.01*** | **0.03*** | 0.68 | 0.38-1.22 | 0.20 | 0.79 |
| cg13079193 | 3 | 8802718 |  | 1.25 | 0.89-1.74 | 0.20 | 0.80 | 1.06 | 0.88-1.28 | 0.55 | 1.00 | 1.09 | 0.73-1.63 | 0.68 | 1.00 |
| cg11589699 | 3 | 8806317 | N_shelf | 1.04 | 0.78-1.38 | 0.79 | 1.00 | 1.14 | 0.86-1.51 | 0.37 | 1.00 | 0.88 | 0.65-1.19 | 0.40 | 1.00 |
| cg00385883 | 3 | 8808259 | N_shore | 0.99 | 0.74-1.32 | 0.96 | 1.00 | 0.97 | 0.79-1.18 | 0.73 | 1.00 | 0.87 | 0.47-1.58 | 0.64 | 1.00 |
| cg03257388 | 3 | 8809213 | Island | 0.88 | 0.57-1.37 | 0.58 | 1.00 | 0.92 | 0.78-1.09 | 0.34 | 1.00 | 0.98 | 0.76-1.27 | 0.89 | 1.00 |
| cg15317815 | 3 | 8809306 | Island | 0.89 | 0.61-1.30 | 0.55 | 1.00 | 1.00 | 0.87-1.15 | 0.97 | 1.00 | 0.94 | 0.70-1.26 | 0.68 | 1.00 |
| cg04523291 | 3 | 8809501 | Island | 0.92 | 0.52-1.61 | 0.76 | 1.00 | 1.00 | 0.85-1.19 | 0.96 | 1.00 | 0.94 | 0.73-1.22 | 0.67 | 1.00 |
| cg02192228 | 3 | 8809536 | Island | 0.83 | 0.47-1.47 | 0.52 | 1.00 | 0.98 | 0.80-1.20 | 0.86 | 1.00 | 1.23 | 0.91-1.66 | 0.19 | 0.74 |
| cg27501759 | 3 | 8809715 | Island | 0.89 | 0.75-1.06 | 0.19 | 0.77 | 1.00 | 0.88-1.13 | 0.97 | 1.00 | 0.89 | 0.74-1.07 | 0.22 | 0.87 |
| cg12695586 | 3 | 8810077 | Island | 0.82 | 0.47-1.43 | 0.48 | 1.00 | 0.87 | 0.66-1.14 | 0.31 | 1.00 | 1.07 | 0.79-1.45 | 0.67 | 1.00 |
| cg19619174 | 3 | 8810139 | Island | 1.19 | 0.87-1.63 | 0.28 | 1.00 | 0.98 | 0.79-1.21 | 0.83 | 1.00 | 0.91 | 0.63-1.30 | 0.59 | 1.00 |
| cg11171527 | 3 | 8810206 | Island | 0.94 | 0.81-1.10 | 0.47 | 1.00 | 0.94 | 0.85-1.05 | 0.30 | 1.00 | 0.94 | 0.79-1.12 | 0.51 | 1.00 |
| cg03987506 | 3 | 8810549 | Island | 1.36 | 0.98-1.91 | 0.07 | 0.27 | 1.14 | 0.91-1.43 | 0.25 | 0.98 | 0.80 | 0.57-1.13 | 0.21 | 0.84 |
| cg00078085 | 3 | 8810592 | Island | 1.00 | 0.84-1.19 | 0.98 | 1.00 | 0.99 | 0.88-1.11 | 0.80 | 1.00 | 1.01 | 0.84-1.21 | 0.94 | 1.00 |
| cg17285225 | 3 | 8811004 | Island | 1.14 | 0.74-1.77 | 0.55 | 1.00 | 0.93 | 0.69-1.26 | 0.65 | 1.00 | 1.42 | 0.93-2.18 | 0.11 | 0.43 |
| cg09353063 | 3 | 8811092 | Island | 1.24 | 0.92-1.68 | 0.16 | 0.63 | 1.11 | 0.90-1.37 | 0.33 | 1.00 | 1.06 | 0.76-1.47 | 0.74 | 1.00 |
| cg23391006 | 3 | 8811279 | Island | 1.17 | 0.76-1.80 | 0.47 | 1.00 | 1.15 | 0.85-1.55 | 0.36 | 1.00 | 0.66 | 0.40-1.10 | 0.11 | 0.44 |
| cg00247334 | 3 | 8811543 | S_shore | 0.75 | 0.58-0.96 | **0.02*** | 0.08 | 0.93 | 0.78-1.10 | 0.37 | 1.00 | 1.28 | 0.99-1.66 | 0.06 | 0.24 |
| cg17036624 | 3 | 8811601 | S_shore | 0.86 | 0.65-1.14 | 0.31 | 1.00 | 0.93 | 0.77-1.13 | 0.46 | 1.00 | 1.04 | 0.77-1.40 | 0.80 | 1.00 |
| cg03710862 | 3 | 8811728 | S_shore | 0.82 | 0.56-1.20 | 0.30 | 1.00 | 0.92 | 0.74-1.13 | 0.43 | 1.00 | 1.01 | 0.74-1.38 | 0.97 | 1.00 |
| cg25085537 | 3 | 8811739 | S_shore | 0.83 | 0.64-1.09 | 0.19 | 0.75 | 0.83 | 0.69-0.99 | **0.04*** | 0.17 | 1.36 | 1.02-1.83 | **0.04*** | 0.16 |
| cg14483142 | 3 | 8811758 | S_shore | 0.81 | 0.64-1.03 | 0.08 | 0.33 | 0.92 | 0.78-1.09 | 0.33 | 1.00 | 1.05 | 0.69-1.61 | 0.82 | 1.00 |

*Chr* Chromosome, *SE* standard error, *N_shelf* north shelf, *N_shore* north shore, *S_shore* south shore
***** Statistically significant p-values (>0.05)
** Information extracted from the Illumina’s Infinium MethylationEPIC v1.0 B5 manifest file. Genomic coordinates are according to the GRCh37 version of the human genome

**Supp. Table 9. Results from association tests between *OXTR* DNAm levels and breastfeeding problems including sore nipples, with adjustment for maternal smoking, maternal age, selection variable (ART), and cell type proportions.**

| **Illumina CpG ID** | **Chr **** | **Position **** | **Relation to CpG island**** | **Model C: Breastfeeding problems** | | | |
| --- | --- | --- | --- | --- | --- | --- | --- |
|  |  |  |  | **OR** | **95% CI** | **p-value (raw)** | **p-value  (adj)** |
| cg26455676 | 3 | 8797459 |  | 0.69 | 0.37-1.27 | 0.24 | 0.94 |
| cg13079193 | 3 | 8802718 |  | 0.91 | 0.73-1.15 | 0.44 | 1.00 |
| cg11589699 | 3 | 8806317 | N_shelf | 0.91 | 0.69-1.21 | 0.51 | 1.00 |
| cg00385883 | 3 | 8808259 | N_shore | 0.87 | 0.68-1.12 | 0.28 | 1.00 |
| cg03257388 | 3 | 8809213 | Island | 1.03 | 0.70-1.50 | 0.89 | 1.00 |
| cg15317815 | 3 | 8809306 | Island | 0.94 | 0.67-1.31 | 0.71 | 1.00 |
| cg04523291 | 3 | 8809501 | Island | 0.98 | 0.77-1.26 | 0.90 | 1.00 |
| cg02192228 | 3 | 8809536 | Island | 1.05 | 0.83-1.33 | 0.67 | 1.00 |
| cg27501759 | 3 | 8809715 | Island | 0.91 | 0.77-1.07 | 0.26 | 1.00 |
| cg12695586 | 3 | 8810077 | Island | 1.04 | 0.82-1.32 | 0.73 | 1.00 |
| cg19619174 | 3 | 8810139 | Island | 0.86 | 0.65-1.12 | 0.26 | 1.00 |
| cg11171527 | 3 | 8810206 | Island | 0.99 | 0.87-1.12 | 0.83 | 1.00 |
| cg03987506 | 3 | 8810549 | Island | 0.89 | 0.64-1.25 | 0.51 | 1.00 |
| cg00078085 | 3 | 8810592 | Island | 1.00 | 0.87-1.14 | 0.98 | 1.00 |
| cg17285225 | 3 | 8811004 | Island | 1.31 | 0.93-1.83 | 0.12 | 0.47 |
| cg09353063 | 3 | 8811092 | Island | 0.99 | 0.76-1.29 | 0.94 | 1.00 |
| cg23391006 | 3 | 8811279 | Island | 0.79 | 0.45-1.38 | 0.41 | 1.00 |
| cg00247334 | 3 | 8811543 | S_shore | 1.15 | 0.93-1.41 | 0.19 | 0.76 |
| cg17036624 | 3 | 8811601 | S_shore | 0.93 | 0.74-1.17 | 0.52 | 1.00 |
| cg03710862 | 3 | 8811728 | S_shore | 1.13 | 0.87-1.47 | 0.36 | 1.00 |
| cg25085537 | 3 | 8811739 | S_shore | 1.30 | 0.91-1.86 | 0.15 | 0.60 |
| cg14483142 | 3 | 8811758 | S_shore | 1.05 | 0.86-1.29 | 0.62 | 1.00 |

*Chr* Chromosome, *SE* standard error, *N_shelf* north shelf, *N_shore* north shore, *S_shore* south shore
***** Statistically significant p-values (>0.05)
** Information extracted from the Illumina’s Infinium MethylationEPIC v1.0 B5 manifest file. Genomic coordinates are according to the GRCh37 version of the human genome

**Supp. Table 10.** **Results from association test between breastfeeding outcomes and an interaction term including *OXTR* DNAm levels and rs53576 genotype with adjustment for maternal smoking, maternal age, selection variable (ART), and cell type proportions. Results are reported for the interaction term.**

| **Illumina CpG ID** | **Model A: Breastfeeding initiation** | | | **Model B: Breastfeeding maintenance** | | | **Model C: Breastfeeding problems** | | |
| --- | --- | --- | --- | --- | --- | --- | --- | --- | --- |
|  | **Log odds** | **SE** | **p-value (raw)** | **Log odds** | **SE** | **p-value (raw)** | **Log odds** | **SE** | **p-value (raw)** |
| cg12695586 | 0.07 | 0.32 | 0.82 | -0.16 | 0.16 | 0.31 | 0.29 | 0.36 | 0.42 |
| cg00078085 | 0.15 | 0.17 | 0.36 | -0.02 | 0.11 | 0.85 | 0.35 | 0.31 | 0.26 |

*SE* standard error

**Full wording of six items used to determine maternal lifetime history of depression.**

Have you ever experienced the following for a period of 2 weeks or more?

1. Felt depressed, sad

2. Had problems with appetite or eaten too much

3. Been bothered by lack of energy

4. Blamed yourself and felt worthless

5. Had problems with concentration or had problems making decisions

6. Had at least 3 of the problems named above simultaneously

**Wording of questions to establish whether mothers experienced any breastfeeding problems**

| **Question** | **Response options** |
| --- | --- |
| Did you go to your doctor/midwife/health visitor for your own health problems during the first month after the birth? | 1 = No, 2 = Yes, ___ times |
| If yes, what was the reason for this? | Perineal wounds/stitches, Caesarean section wound, Mastitis, Sore nipples, **Breastfeeding problems**, Other, specify: ___ |
